# From Envy to Radicalization

**DOI:** 10.1007/s40806-023-00380-1

**Published:** 2023-11-29

**Authors:** Michael Moncrieff, Pierre Lienard

**Affiliations:** 1 Department of International Public Law & International Organization, University of Geneva, Geneva, Switzerland; 2 Department of Anthropology, University of Nevada, Las Vegas, NV USA

**Keywords:** Radicalization, Envy, Extremism, Violence, Terrorism, Functional model

## Abstract

Models of radicalization have typically placed grievances at the heart of radicalization. In contrast, we argue that viewing the radicalizing agent as decidedly *proactive*, and less reactive, better accounts for the available data. At the core of our radicalization model is the functional structure of envy. The operative properties of the emotion align with essential and conspicuous features of the radicalization process: a motivation to monitor social differentials, an identification of sources of postulated welfare costs, an impulse to eliminate or depower purported competitors, an attempt to diffuse responsibility for one’s aggressive actions, and the rejoicing at the envied agent’s misfortune. Two of those operative properties are particularly important for our understanding of radicalization. Envy motivates the neutralization of competitors when responsibility for welfare costs is not objectively attributable to others’ wrongdoing toward the party who feels injured. The “process of typification” serves as a means to diffuse responsibility. It extends the reach of individual concerns by downplaying the particulars of the personal situation motivating the envious agent while evoking universally shared interaction templates (e.g., humiliation, injustice) to appeal to a broader audience.

Between 2012 to 2016, approximately 4,000 citizens from large multicultural urban centers of the European Union decided to join militant groups in Syria (van Ginkel et al., 2016). Those individuals chose to do so of their own volition and, in the process, exposed themselves to tremendous risks. The voluntary nature of their participation appears perplexing, given the inherent collective action problem involved (Olson, 1965)—it would be far safer to abstain from participating while reaping the benefits of others’ militancy since non-contribution could hardly be sanctioned. What drives individuals toward a radicalization with such inherent risks?

A widely adopted analytical perspective places the emergence of a commitment to intergroup conflict at the core of radicalization, signifying a change in attitude and beliefs about intergroup violence and self-sacrifice (McCauley & Moskalenko, 2008, p. 416). Despite extensive research, a definitive consensus on the exact nature of radicalization remains elusive (Coolsaet, 2019; Neumann, 2013). We adopt a narrower conceptualization of radicalization, focusing on its occurrence within contemporary “welfare” states, particularly Western liberal democracies. Within such contexts, radicalization entails forsaking relative peace and safety for ventures marked by high-risk entrepreneurship. While it is acknowledged that phenomena resembling “radicalization” may manifest in other contexts, such as genocides and mass killings (e.g., Mcdoom, 2020), we remain agnostic about their similarities. Two propositions seem to constitute the core architecture of the radicalized mind (see Lienard & Moncrieff, 2023): (1) for the radicalized agent, action is invariably deemed optimal, strictly preferred over inaction, and (2) the costs incurred due to one’s bold actions warrant entitlement and recognition.

The classic account of radicalization’s transformative process found in the literature specifies a cluster of diagnostic constituents (e.g., Borum, 2003, 2011a; Hafez & Mullins, 2015; Horgan, 2005; Moghaddam, 2005; Sageman, 2008; Silber et al., 2007; Wiktorowicz, 2005), including:

1. Some *individual susceptibility* associated with negative life experiences and adversity or an otherwise negative appraisal of one’s condition and a desire to improve one’s situation.
2. A *displacement of aggression* toward agents judged responsible for one’s grievances (e.g., person, group, nation).
3. And a *collectivization* of concerns involving the association with a radical social network or ideology promoting a specific moral project, violence, and a cohesive whole.

Most causal theoretical radicalization models converge on a similar set of prominent constituents (Gøtzsche-Astrup, 2018).

Radicalization is conjectured to originate in grievances about perceived injustice, inequality, relative deprivation, discrimination, ostracism, and alienation (e.g., Abbas & Siddique, 2012; Ajil, 2019; Baugut & Neumann, 2019; Bloom, 2006; Botha, 2014; Della Porta, 2013; Doosje et al., 2013; Florez-Morris, 2007; Horgan, 2004; McCauley & Moskalenko, 2011; Orsini, 2013; Pedahzur, 2005; Post et al., 2003; Sageman, 2004, 2008, 2017b; Scorgie-Porter, 2015; Stern, 2003; Webber et al., 2017) and to the social emotions evoked in such circumstances such as outrage, indignation, shame, guilt, humiliation, and a desire for revenge (e.g., Bloom, 2006; Cottee, 2011; Ilardi, 2013; Kriner, 2018; McCauley, 2017; Merari et al., 2009; Pedahzur, 2005; Pisoiu, 2015; Sageman, 2017b; Speckhard, 2016; Stern, 2003; Webber et al., 2017). The transformative process would spring from an entanglement of constraints to which the radicalizing individual *reacts*. Other models hold at their core the social benefits gained from cooperation as the critical catalysts of radicalization, including such uplifts as affiliation to groups thanks to the sharing of beliefs and a common ideology, social significance rewards, and reputational enhancement (Goldman & Hogg, 2016; Hogg, 2014; Hogg & Adelman, 2013; Webber et al., 2018).

Our model of radicalization puts at its center a *proactive*—instead of reactive—agent (Lienard & Moncrieff, 2023; Moncrieff & Lienard, 2021, 2023). We argue that the emotion of envy—an emotion that motivates individuals to monitor their surroundings, assess the prosperity of others, and seek the eradication of the status divergence—plays a key role in radicalization. Moreover, we hold that the operative properties of the emotion—evaluative, attitudinal, behavioral, and belief-altering—determine the typical evolvement of the radicalization process.^1^ It will be shown that a functional account of envy’s role in radicalization unifies the findings of various theoretical models under a single explanatory framework, including the observations that, seemingly, radicalized agents recur to violence to achieve social significance (Quest for significance*,* Webber & Kruglanski, 2018), seek out radical group affiliations for their emotional benefits (Uncertainty-identity theory, Hogg & Adelman, 2013), and are driven by ideological conviction (Devoted Actor Model, Atran, 2016); while radicalized beliefs would still be understood as orthogonal to violent actions (Two-pyramid approach, McCauley & Moskalenko, 2017). Moreover, our functional account of envy’s role will make clear why violence should be expected in radicalization.

Whereas other theories such as *relative deprivation theory* (Gurr, 2015; Kunst & Obaidi, 2020; Moghaddam, 2005) and *social identity theory* (Goldman & Hogg, 2016; Hogg, 2014; Hogg & Adelman, 2013) approach radicalization at a psychological, i.e., *intentional* level, our model is at a more fundamental level, a computational and *functional* one (Pietraszewski & Wertz, 2021). At the functional level, emotions are viewed as superordinate programs synchronizing the functioning of subprograms or “subcomponents” in charge of various functions (e.g., perception, memory, and motivations) that would be necessary for the deployment of evolutionarily adaptive responses (Cosmides & Tooby, 2000; Tooby & Cosmides, 1990, 2008, p. 117).

The distinction between functional and intentional levels (see, Pietraszewski & Wertz, 2021) can be illustrated with relative deprivation theory applied to explain radicalization*.* The theory describes how social comparison, the appraisal of disadvantage, feelings of unfairness and entitlement, and subsequent emotions such as anger and ressentiment (Smith et al., 2012) account for violent extremism (Gurr, 2015; Kunst & Obaidi, 2020; Moghaddam, 2005). The theory makes a connection between social constraints, subjective assessment, and mental states. However, it does not attempt to explain what gives rise to such intentional level mental states (e.g., sense of unfairness, anger, ressentiment) nor makes any precise predictions about the computational systems that would cause such mental states (i.e., the input/output, if/then rules). The absence of a lower-level computational description is apparent in criticisms of the theory, e.g., “predicting whom members of a group will select for purposes of comparison, and under what circumstances, remains a fundamental issue for relative deprivation” (Moghaddam & Taylor, 1994, p. 135). Indeed, it is noteworthy that Gurr (2015) and subsequent scholars using relative deprivation to explain radicalization never mention envy (e.g., Borum, 2003; Moghaddam, 2005). Refocusing on envy, one may decide to describe the feeling of envy as a mental state that moves people to particular behaviors. Although a legitimate perspective, it would still not explain the function that the emotion fulfills. In other terms, what has envy been designed to perform and, consequently, what it leads to?

The present study explores the operational tasks necessary for the effective functioning of envy and identifies key information-processing functions central to this process. Subsequently, these functions are compared with the principal components that characterize radicalization. It is important to acknowledge that this investigation does not present a comprehensive or computationally exhaustive explanation of envy’s role in radicalization. Indeed, compared to other emotions, envy is meagerly studied. Our primary objective is to underscore envy’s significance in the context of radicalization to prompt further research, discussion, and debate. Moreover, we seek to stimulate additional inquiry into the functional structure of envy.

## The Elemental Processes of Envy

We argue that envy functions to motivate the agent to track the advantages others are perceived to have, to be sensitized to the purported fitness-suppressing consequences those might have for the envious individual, and to attend to the eradication of the differential (e.g., Fiske, 2011; Hill & Buss, 2008; Moncrieff & Lienard, 2023; Schoeck, 1966; Smith & Kim, 2007; Sznycer et al., 2017).^2^ It is crucial to delineate the difference between envy and jealousy, as these two emotions are often mistakenly used interchangeably. Envy and jealousy are distinct emotional experiences, each serving different functional roles (Leahy, 2021; Parrott & Smith, 1993). Specifically, jealousy instills vigilance and safeguarding actions toward relationships of value, especially when such relationships are threatened by third-party intrusion (Buss & Haselton, 2005; Yong & Li, 2018). Furthermore, our conceptualization of the emotion of envy should not be confused with the colloquial understanding of the term. We are not interested in the qualia of the emotion nor its common social psychological conception, but instead, its evolved function. The elucidation of the logic of envy’s structure, organization, and operation—what it does and how it does it—requires adopting a functional perspective.

We propose a novel conceptualization of envy’s role in radicalization. As we elaborate in “Connecting the Proposed Elemental Processes in the Radicalization Literature to Envy” section, the principal components that characterize radicalization—individual susceptibility, displacement of aggression, and collectivization—closely match subcomponents or elemental processes that capture the logic of the initial course of envy—we qualify them as motivational, identificational, attributional, and diffusional.

The *motivational* component of envy promotes the monitoring of potential welfare risks, as revealed by conditional proxy cues of social differentiation. The motivational feature of envy is best understood in the context of the human propensity to consider many human interactions, social goods, and accesses to different resources as fundamentally zero-sum^3^ (i.e., circumstances in which one’s gain is interpreted as another’s loss) (Foster, 1965; Gershman, 2015; Hill & Buss, 2008; Lin & Bates, 2021; Schoeck, 1966; Smith & Kim, 2007; Sznycer et al., 2017). Enduring lasting ostracism in a world of scarcity, the world of our evolutionary past, would have been potentially fatal, hence the importance of having a strong intrinsic motivation to monitor the environment for cues of potentially threatening social differentiation (Hill & Buss, 2008). Consequently, envy tracks how others within one’s social environment value certain characteristics, actions, or possessions (Landers, 2023; see also, Sznycer & Lukaszewski, 2019). Envy is heightened when the valuation of a rival competitor’s characteristics increases in the eyes of a relevant audience (i.e., individuals whose valuation the envier perceives to matter to his fitness) (Landers, 2023). This contrasts with previous research positing that envy is driven by what the envier desires or his similarity to the envied (see Smith, 2004). Furthermore, the elicitation of envy is sensitive to the divisibility of resources: envy is more strongly experienced when competitive resources are more easily divisible but not expected to be shared (Inoue et al., 2015).

The *identificational* moment comprises various assessments. Can the evidence of the social distinction be traced to deeds, behaviors, or observable attributes of a single individual, a number of them, or a collective? When an individual possesses a distinctly enviable object or characteristic, envy is most likely directed at that particular individual (see, Vendrell Ferran, 2021). Envy may be directed toward collectives or social categories when members are perceived to possess similar enviable properties. Moreover, shared attributes prompting agents to perceive one another as interchangeable may also engage psychological systems specialized for reasoning about coalitions (Pietraszewski, 2016). The identification of the envied target yields notable effects. Envy enhances attention to potential targets and memory for information about them (Hill et al., 2011; Zhong et al., 2013). When envy leads to hostility, attention is biased away from means to improve one’s situation and toward the envied target rather than its superior fortune (Crusius & Lange, 2014). In such situations, counterfactual thinking about envied targets—considering what one could have done differently to obtain what one does not possess—is also reduced (Crusius & Lange, 2021).

The identificational moment also relates to internal assessments probing the “deservedness” of the advantage—how justified is the difference?—with its typical concomitant questions—have I previously benefited, or will I plausibly benefit from that individual or group in the future? Those questions should be understood as high-level formulations of low-level functional computations, which means that the agent does not have to be aware, even in part, of the various ongoing assessments of aspects of the situation being analyzed. It is important to note that others might reason that some advantage is undeserved for reasons other than envy (Landers & Shaw, n.d.). When high achievers are presented as undeserving of their success, it increases others’ negative feelings toward them (e.g., Van de Ven et al., 2012). However, these negative evaluations might not stem from envy but rather from motivations related to morality or fairness (Landers & Shaw, n.d.). Such discrepancies afford an opportunity for an envier to conceal their envy by aligning with the moral sensibilities of others. The potential for diffusing responsibility among others (Sznycer et al., 2017), the ability to execute spiteful actions covertly (Cikara & Fiske, 2012), and the respective formidability of the envier and envied (Sell et al., 2009) are additional factors influencing whether it is prudent to act upon envy.

The *attribution of responsibilities* is our next elemental process. We argue that envy may be activated in situations where the attribution of *direct* responsibility for perceived welfare costs is impossible to make or, at best, equivocal. Indeed, when an agent perceives another’s direct involvement in wrongdoing, other emotions are expected: anger, hatred, fear… not envy. The perceived depression of welfare imputed to the source does not need to be linked to any directly nefarious actions toward the envious individual. This is evidenced by the fact that once a negative appraisal has occurred, envy does not require any physical interaction or overt competition to activate its downstream effects (i.e., pleasure at the misfortune of the envied); mere stereotypes are enough (van Dijk et al., 2015). In sum, envy is a proactive emotion not requiring others’ harmful actions to be activated.

We now turn to the *outputs of envy*. If the outline of the superordinate program of envy presented here is correct, the expected consequence of envy should be a desire to reduce the welfare of the better-off (Sznycer et al., 2017). Thus, the evolved function of envy would be to motivate the neutralization of superior resource competitors^4^ even in absentia of the easy and *direct* imputation of responsibility for presumed welfare costs. The literature is rife with examples of the link between envy, hostility, and aggression (Miceli & Castelfranchi, 2007; Morgan et al., 2022; Smith & Kim, 2007), even when the imposition of costs on competitors entails harm to the envious individual (Wobker, 2015; Zizzo & Oswald, 2001). It is well established that enviers get pleasure from the injury and suffering of the envied (i.e., schadenfreude) (Smith et al., 1996; Van de Ven et al., 2015), envy reduces empathic responses to others’ misfortunes (Chester et al., 2013), and it can lead to “moral disengagement” (Rengifo & Laham, 2022). Envy is also shown to activate other self-enhancing emotions such as pride, and pride displays reciprocally enhance one’s experience of envious feelings (Lange & Crusius, 2015). Greater impulsivity is also linked to the expression of increased envy-driven behaviors (Crusius & Mussweiler, 2012) and envy lowers one’s ability to engage in acts requiring greater volition (Hill et al., 2011).

Envy is known to motivate the masking of spiteful actions using stealth (Schoeck, 1966), which lends to envy’s “secretive nature” (Heikkinen et al., 2003; Hill & Buss, 2008; Smith & Kim, 2007). Being unprovoked, the envy-motivated attack is regarded as indefensible.^5^ It has the additional drawback of lending itself to being diagnosed by onlookers as indication of the enviers’ readiness to victimize others wrongfully. Not surprisingly, such an attack is bound to elicit a strong moral condemnation (Pietraszewski, 2016, p. 475). Furthermore, the envious agent’s obvious disregard for potential gains and steep discount of likely welfare costs, so long as it decreases the target’s welfare, communicate unwanted traits in social partners (Barclay, 2013). Those attitudes and decision choices typical of the envier may also signal that the agent lacks genuine moral restraints, thus calling into question the envier’s trustworthiness (Frank, 1988, 2004). People go to great lengths to avoid being the target of envy given the threat it poses (Foster et al., 1972; Parrott & Rodriguez Mosquera, 2008; Rodriguez Mosquera et al., 2010; Van de Ven et al., 2010). Such impulsive social agents should also be judged as less reliable, marking them as improbable partner choices, given the increased risk that one’s association with them generates (Baumard et al., 2013). To envy’s reputational cost, Silver and Sabini (1978) add the injure to the envier’s social value, making envy “doubly damaging” if unmasked. Envy discloses more than what its experiencer would want to, as it indirectly signals that the *coveted* is not *owned.* That information devaluates how desirable the envier is as a potential partner, which, in turn, drives social agents to downregulate their inclination to tradeoff some of their welfare for that individual’s (Sznycer et al., 2016).^6^ Social shunning has involved significant fitness costs throughout human evolutionary history. We should expect a strong evolved disposition to address the threat of social devaluation (Sznycer et al., 2016), which should comprise a proneness to conceal the envious motives behind one’s decisions and actions. Indeed, enviers typically hide their experience of the emotion from others (Smith, 2004). Fascinatingly, envy lacks a distinct facial expression or bodily display (Miceli & Castelfranchi, 2007; Smith & Kim, 2007), facilitating concealment.

The ambiguity of one’s envious motivations allows the individual to manipulate others’ perceptions to his advantage (Hill & Buss, 2008). We argue that another output of envy—*diffusion*—should entail the search to diminish one’s exposure to potential retaliation for one’s actions against the envied by attempting to diffuse responsibility among others, bringing them into a coalitional reaction to the envied individual(s) (Sznycer et al., 2017). Indeed, there is some evidence in the literature that envy may underly coordination and cooperation (Fiske, 2011; Gros, 2021; Vignolo, 2005). Envy is inherently motivated by self-interest. However, if the benefits of collective action are to be realized—the diffusion of responsibility, divisions of costs, benefits of coordination power (see Hardin, 1995)—self-interest must be suppressed (or appear suppressed) in favor of the collective interest. The unveiling of self-interest can quickly destroy the coordination and commitment necessary for successful collective action (Olson, 1965; Price et al., 2002). To resolve this dilemma, collectives may generate common knowledge of sacred narratives (i.e., ideologies, beliefs, moralities) or collective fictions that reframe group-serving activities as benevolent and help to establish coordination norms, deviations from which can lead to condemnation, punishment, or exclusion (Bang Petersen et al., 2021; Marie & Petersen, 2023; Pinsof, 2023).

The elemental processes proposed above attempt to clarify what transpires early in the course of the emotion, which, as we will see, has consequences on how we should understand the process of radicalization.

## Connecting the Proposed Elemental Processes in the Radicalization Literature to Envy

Is there evidence of an unambiguous connection between envy and extremism? Indeed, recent studies have shed light on the connection between envy and extremism^7^. Moncrieff and Lienard (2023) found that envy significantly predicts core aspects of radicalization, including endorsing extremism and accepting violent means to achieve one’s ends. Additional research supports the positive correlation between envy, core aspects of radicalization, and impulsivity (specifically negative and positive urgency) (Moncrieff & Lienard, n.d.). Envy emerged as a mediator in the relationship between negative urgency and radicalization, suggesting that for impulsive individuals, envy-driven motivations may hasten the neutralization of perceived social disparities, thus fueling the radicalization process (see “Motivational” section).

The link between envy and radicalization also aligns with the literature on lone shooters. Available information about one of the Columbine High School shooters makes clear that he was not socially isolated, teased, or bullied but was liked and involved in social activities. Yet, his journal entries underscore how envy thoroughly urged him on (Langman, 2009, p. 53). Some perpetrators, self-aware enough to fully grasp their envious motivation (p. 150), openly justified their actions as meeting their desire to eliminate other social competitors’ advantages by pursuing an alternative path of reputation enhancement to the usual routes available in school settings.

In the midst of other motivators, envy has also been identified as one motivation behind other types of violence outside of educational settings, including mass murders, hate crimes, and other racist violence (e.g., Knoll & Meloy, 2014; Meloy et al., 2004; Myketiak, 2016). Envy appears to lead to an increase in the frequency of hate crimes (Gale et al., 2002). Greater parity between black and white incomes—in the specific context studied by Gale et al. (2002), an increase in black households’ income relative to white households’—was associated with higher white-on-black hate crimes, while the overall crime rate decreased. Qualitative research has also found envy to be involved in racist violence, with one article noting “both shame and rage are dangerously present in perceptions of Asians as powerful and successful, and thus objects of envy […]” (Ray et al., 2004, p. 363). Fiske (2011, p. 130), too, makes a strong case for the role of envy in motivating violence against members of successful entrepreneurial ethnic groups.

Envy is sometimes mentioned in the literature on terrorism and violent extremism (e.g., Pipes, 1992; Trip et al., 2019; Vetlesen, 2006), but rarely as prime mover *in isolation* from other emotions. Cottee (2021) somewhat departs from the general trend by briefly insisting on the potentially central contributing role that envy could have in incel-inspired terrorism.^8^ Incel refers to an online subculture promoted by men who, incapable of matching the demands of the mating market, find themselves stuck into *in*voluntary *cel*ibacy (Costello et al., 2022; Hoffman et al., 2020). Indeed, envy is repeatedly mentioned in the manifesto of a member of the incel online subculture who violently attacked members of the public (Rodger, 2014).

Although the scholarly focus seems to be more surely grabbed by hints of emotions such as shame, humiliation and indignation (e.g., Cottee, 2021; Hoffman et al., 2020; Juergensmeyer, 2017, p. 241), envy appears to creep back in, here and there in the literature. Why such a systematic downplaying of envy? Why privileging reactive or “*defensive*” emotions (e.g., humiliation, indignation) over a proactive or “*offensive*” one (i.e., envy)? We argue in the “Identificational and Attributional” section that the deemphasizing of envy’s role in radicalization studies might be traced to the very nature of the emotion prompting its concealment and the strong intuitiveness of the retaliatory action template. As shown in the following sections, despite the lack of direct references to envy in the literature on radicalization, many of the features of radicalization can be accounted for by an envy-based reading of the evidence. Table 1 highlights some significant commonalities between envy and characteristics of radicalization found in the literature.

**Table 1:** Comparing envy to elements of the radicalization phenomenon

**Envy**	**Characteristics of radicalization**
**Precipitant**	
Judgment by the individual that there is a zero-sum resource competition	Black-and-white/zero-sum conceptualization of social world (van den Bos, 2020)
**Potential threat**	
The risk of a lasting exclusion from benefits (e.g., prestige, status)	The risk of being ostracized in a manner consequential to welfare (Pfundmair, 2019); exclusion from mating market (Cottee, 2021)
**Goal**	
To neutralize superior resource competitors in situations where *direct* responsibility for welfare costs cannot be immediately attributed to others	Dedication to aggressive action to eliminate the individual(s) responsible for the discrepancy (Baele et al., 2021)
**Motivations**	
To identify the source (individual, set of individuals, or collective) that responsibility may be attributed to	Identification of class of individuals because of their existence *e.g.,* hatred toward Blacks, Jews, and Hispanics (Ware, 2020)
To derive pleasure at the misfortune of the envied	Exhilaration and sensation seeking for aggressive actions against the hated category of individuals (e.g., Stern, 2014; Wolfowicz et al., 2019)
**Actions and behaviors**	
To (spitefully) reduce the welfare of the better off	Willingness to take great risk for acting and radical rejection of the status quo (Saucier et al., 2009)
To avoid devaluation by concealing the emotions from others	Obvious desire to achieve reputational boost (Kruglanski et al., 2019) and masking of selfish motivations (2019, p. 17)
To diminish one’s exposure to potential retaliation by diffusing responsibility among others	Searching for coalitions (McCauley & Moskalenko, 2017) and ideologies / violence justifying narratives (Kruglanski et al., 2019)
**Modulating factors**	
Individual differences (e.g., impulsivity, age, IQ, personality)	Impulsivity, and youth and sex of the of the radicalized agents (Wolfowicz et al., 2019)
Social environment characteristics (e.g., marked division between in/outgroup, class distinctions)	Divided ethnic or religious sociopolitical milieus (Franz, 2015; Jacobson & Deckard, 2012; Rabasa, 2014)

## Connecting Components of the Model to the Radicalization Literature

### Motivational

The motivational aspect of envy, which promotes the monitoring of potential welfare risks, closely matches the first principal component of our radicalization characterization—individual susceptibility*.* The negative appraisal of one’s condition associated with this component of radicalization matches what we would expect as zero-sum social comparisons play a crucial role in engendering envy (Alicke & Zell, 2008; van de Ven & Zeelenberg, 2020). Characteristics of the social environment and individual differences that impact how easily individuals are prone to experience envy should also be linked to an increased risk of radicalization.

Neutralizing one’s competitors might be achieved in different fashions: competition, avoidance, submission, or aggression (Fiske, 2011; Hill & Buss, 2008). The choice of approach taken rests on complex cost–benefit computations involving the assessment of plausible success, possible social support, exit options, etc. When does envy become a salient option? Three primary dimensions need to be taken into account: (1) the complexity of the advantage, (2) the traits and dispositions of the individuals in the envious dynamics, such as IQ, psychological traits (e.g., impulsivity, age, skills), and (3) the nature of the socio-cultural context (e.g., a social world where agonistic or hierarchical norms determine expected behavior) or the characteristics of the socioscape (e.g., marked division between in/outgroup, class distinctions). We argue that the greater the intractability, the more salient envy should become (Protasi, 2016). Why? Because those contextual features and differentials raise the cost of competing, matching the opponent’s strength, or avoiding the competition altogether. Thus, when the costs of matching the competition are construed as too steep, its elimination becomes increasingly tempting as an expedient approach. In conditions of great uncertainty, the impulsive choice of a shorter-term benefit of lesser lasting value should quickly become the preferred option. Indeed, envy has been linked to impulsivity (e.g., Crusius & Mussweiler, 2012; Hill & Buss, 2006). Envy leads to the choice of the impulsive option cutting through uncertainty by favoring greater short-term visibility. This may account for why social ostracism is empirically implicated in the process of radicalization (Knapton, 2014; Pfundmair, 2019; Pfundmair & Wetherell, 2019) as ostracism evokes zero-sum competition but constrains one’s choices for neutralizing competitors.

Actions and characteristics that are valuable in one social environment (e.g., impulsivity and aggression in prison) may not be valuable nor socially valued in other environments (e.g., impulsivity and aggression in school) (Sznycer & Lukaszewski, 2019). Previous research has described how prestige-based strategies differ from dominance-based strategies (Cheng et al., 2013; Henrich & Gil-White, 2001; Maner & Case, 2016; Petersen et al., 2021; Von Rueden et al., 2011). In social ecologies characterized by prestige-based hierarchies, social recognition is acquired based on service and merit. In social ecologies replete with dominance-based hierarchies, status is more likely to be acquired through coercion and the outcome of zero-sum conflicts. Scholars have acknowledged the connection between status attainment and dispositional envy (Lange et al., 2018), noting how the characteristics of a social ecology may nudge individuals toward chronic prestige or dominance strategies. Lange and Crusius (2015) found that individuals who tended to pursue a dominance strategy had an increased inclination to experience malicious (hostile) envy. Such reasoning may be why envy is negatively correlated with emotional intelligence (Xiang et al., 2017, 2020) but positively correlated with narcissism, Machiavellianism, and psychopathy (Milić et al., 2022).

We argue that divided ethnic or religious sociopolitical milieus, which are conducive to radicalization (Franz, 2015; Rabasa, 2014), are so because they contribute to making envy a salient strategy. Social environments characterized by constraints, scarcity, and inter-coalitional cleavages are more likely to evoke zero-sum thinking and the associated envy, which seeks to level the playing field when differences are perceived. In such a socioscape, internal competition for welfare-enhancing alliance building that allows access to social resources is a prominent feature, which may partially account for Sageman’s (2008) “bunches of guys” radicalization hypothesis. Such logic may also account for why Jacobson and Deckard (2012) found that social characteristics typical of fractionalized tribal systems—hostility toward central third-party institutions, nested grievances and feuding, and corruption—are particularly conducive to the emergence of violent extremism. Modern liberal democracies have several features that generate conditions fueling the saliency of envy (e.g., merit-based opportunities for success, a market economy that lead to enduring differentiations of the social world, an egalitarian ethos, absence of highly visible drastic leveling mechanisms). In addition, recent immigration with the generation of unassimilated cultural clusters creates stark divisions, which ease the stereotyping of groups. A meta-analysis of radicalization risk factors found that a low degree of integration within the wider society combined with a strong identification with a minority identity significantly affected the likelihood of holding radicalized attitudes and engaging in violent actions (Wolfowicz et al., 2019). Indeed, in a sample of European neo-jihadi terrorists, Bakker (2011) found that the families of approximately 92% of those were of extra-European origin (i.e., the neo-jihadists were first, second, or third-generation migrants).

### Identificational and Attributional

The identificational and attributional aspects of envy entail linking the envier’s negative emotions to a target when the attribution of *direct* responsibility for perceived welfare costs is impossible to make, i.e., the target of aggression is blameless. These aspects of envy perfectly match with the displacement of aggression (e.g., Moghaddam, 2005)—where one’s negative feelings are attributed to a perceived causal agent (e.g., person, group, nation) responsible for one’s grievances—that characterizes radicalization. However, whereas envy is noticeable in accounts of individual radicalization, it is absent in much of the expert literature on collective radicalization. We account for the commonly mentioned emotional experiences (e.g., revenge, shame, guilt, humiliation) and the grievances used to explain their evocation in a different manner from previous scholars. Radicalized agents have an inherent incentive to avoid unmasking their self-interested envious motives. To reduce the risk afforded by the unmasking of envious motives, the envier must reframe how others would perceive his actions. There are two primary ways that individuals could make motivations and actions appear less self-interested. The first involves framing the aggressive impulse as defensive rather than offensive (De Dreu & Gross, 2019; Lopez, 2017, 2019; Pietraszewski, 2016). The other rests on the diffusion of responsibility within a plurality of individuals (Sznycer et al., 2017), which will be discussed later under “Diffusion.”

An oft-shared assumption by scholars studying radicalization is that it is possible to infer the causes of such process from the rationalizations provided by radicalized individuals. Such a position rests on the intuition that the rationalization of an action is its explanation (Davidson, 2001, p. 8). That assumption fares less well for the purpose of reaching explanations with strong scientific validity. Mercier and Sperber (2017) emphasize that reasons are for consumption: they have an audience. Thus, justifications of attitude and behavior are unlikely to retrace the actual motivational processes that elicited them. As such, the militants’ explicit rationalizations might obscure the motivations early at work in radicalization—i.e., *envy*. While stealthily concealed, the underlying envious motivations are hinted at by considering why thrill-seeking and excitement are associated with radicalization.

Successful offensive aggression confers rewards to those who partake (e.g., resources acquisition, status increase, reproductive access), while a defensive action produces beneficial collective consequences that are typically non-excludable goods (e.g., safety, deterrence) (Durham, 1976; Glowacki & Wrangham, 2013, 2015; Lopez, 2017, 2019; Pietraszewski, 2016; Tooby & Cosmides, 1988). Differences in these payoffs have shaped our psychology in predictable ways. Offense would primarily activate neural circuitries involved in the processing of rewards, energizing behavioral activation (De Dreu & Gross, 2019). A freely chosen offensive aggression commands self-interest and, therefore, reports of intense pleasure, enjoyment, and excitement for the highly rewarding experience. Interestingly, radicalized individuals report the great excitement (and other similar emotions) that their aggressive militancy generates for them (e.g., Bartlett & Miller, 2012; Cottee & Hayward, 2011; Haggerty & Bucerius, 2018; Jeurgensmeyer, 2001; Mastors & Siers, 2014; Schumpe et al., 2018; Stern, 2014). A meta-analysis of radicalization risk factors found that risk-taking and thrill-seeking were strongly associated with violent engagement (Wolfowicz et al., 2019). Such findings support the proposition that radicalized individuals find the prospect of offensive action quite rewarding, which seems to be part of what motivates them.^9^ Such evidence conflicts with the reasons most often provided to justify the individual’s radicalized beliefs and militant actions. Ajil (2019, p. 17) notes the care that some radicalized individuals take to downplay the reporting of excitement: “It’s all very exciting, being in these situations. […] But it shouldn’t be violence just for the sake of it. […] If you are just guided by your own desires, it merely becomes another manifestation of self-centeredness.” Emotions that signal self-interest should not be given as reasons to justify militant actions. Such reasons should instead focus on reframing offensive motivations in terms of defense. The evocation of social grievances and associated emotional templates is better suited for manipulating others into accepting the legitimacy of one’s decisions and actions.

Harm is more justifiable when it is motivated by defense. We should therefore expect the envier to provide reasons which cue the inputs of emotions designed to motivate agents to respond to harmful actions of others. Indeed, the “retaliation” conceptual template comes equipped with built-in intuitions about the rationale for action, the risk of omission, the inherent benefits of the negative reciprocity, and the praiseworthiness, or, at the very least, the acceptability of the aggression *in return*. In short, “retaliation” provides an immediate grasp of the incentives accounting for the observed behavior. Humiliation is another particularly good example. Scholars studying violent extremism have ascribed a prominent role to humiliation in the radicalization process (Kruglanski et al., 2014; McCauley, 2017; Webber & Kruglanski, 2018). Elison and Harter (2007, p. 314) conceptualize humiliation as an event where an individual experiences a highly intense emotional reaction to having been lowered in the eyes of others because of *someone else’s* purposeful and hostile actions. Typical reactions to humiliation include violent ideation, and (desire of) revenge or retaliation (Elison & Harter, 2007). Gilbert (2019) notes that humiliation is distinct in that it involves an external attribution, a liable hostile other, a feeling of unfairness, and a motivation to look for revenge or retaliation. Humiliation thus immediately evokes in others a template that *someone else’s* purposeful and hostile actions are to blame for any subsequent harmful actions by the actor. That humiliation is commonly used to strategically and rhetorically reframe offense as defense rather than actually *cause* violence may account for Ginges and Atrans’s (2008) finding that the experience of humiliation did *not* contribute to political violence. The humiliation frame makes possible the hiding of brute self-interest behind a collective pretext, which eases the manipulation of others into believing that violent action is necessary and justified. Indeed, radicalized individuals often appeal to others not with their own personal accounts of victimization, but with collective accounts of harms caused by an enemy. Numerous accounts of collective humiliation and harm are found in the literature on radicalization (e.g., Ilardi, 2013; McCauley, 2017; Silke, 2008; Sonpar, 2008; Webber et al., 2018).

### Diffusion

Militants regularly frame violent actions as necessary for the defense of collective goods, values, and safety. Bosi and Della Porta (2012, p. 379), for instance, observe that the mobilization into armed groups and the commitment to violence are most ordinarily justified by the need to defend one’s community (Ajil, 2019, p. 13). Scholars note how some militants also saw “jihad as an individual obligation of the highest order. Seen within the context of self-defense […]. One individual [...] explained that he [recruited fighters] ‘because it doesn’t make sense not to […] you have to defend your family, you have to defend your friends, and your fellow people, to protect them […]’” (Ilardi, 2013, p. 722). The prominence of such justifications in the interviews of militants and other radicalized individuals has convinced scholars that such collective motives play a central role in the explanation of radicalization (Małysz, 2020; O’Gorman & Silke, 2015; Pape, 2006, p. 184). Why would we find such a systematic insistence on a collective duty in the account of radicalized individuals? It has to do with the specific interpretive frame that those reasons generate.

We operationalize the concept of typification as the modeling of a personal plight as a particular case (token) of a prototypical collective situation (type) at the heart of the persuasion process attempting to diffuse responsibility. Seeking out partners to reduce the costs afforded by indulging in envy-driven and spiteful behaviors might explain the data presented in the causal models typically inspired by Social Identity Theory that seek to explain radicalization (e.g., Goldman & Hogg, 2016; Hogg, 2014; Hogg & Adelman, 2013). Rather than identity being a cause of radicalization, it may be that aligning with others who might share common envious interests makes strategic sense. The extent to which one can align with others to pursue envious goals helps to diffuse responsibility (Sznycer et al., 2017) while it increases one’s relative power and formidability (Hardin, 1995; Tooby & Cosmides, 2010). The idea that the possibility of diffusing responsibility might underly one’s willingness to engage in spiteful actions is supported by recent research showing that violent extremist intentions were strengthened when it was perceived that one’s peers had similar attitudes (Kaczkowski et al., 2020). That “lone wolves”—those who attack without the direct support of others—are typically “connected” to a community in some fashion speaks to the facilitating role that the perceived diffusion of responsibility plays in the acting out of radicalized individuals (Sageman, 2017b, p. 6; Schuurman et al., 2019). The fundamental difference between loners and militants belonging to a collective might just be that the loners have not actually found an active collective, ready to act in a concerted fashion. There is evidence indeed that “loners” attempt to anchor their acts into imagined genealogies of perpetrators (e.g., Langman, 2018; Lankford, 2018).

Some scholars have focused on the adoption of beliefs, values, and ideologies—sets of beliefs and values—as being key to understanding why an individual would become radicalized to the point of engaging in extreme and violent action for a collective. The “devoted actor” hypothesis proposes that militant actors are motivated by their adherence to sacred and transcendent values (Atran, 2016; Sheikh et al., 2016). Research on militants supports the fact that many hold strong religious or ideological convictions (e.g., Hoffman, 1995; Perliger & Pedahzur, 2016). In our argument, such non-negotiable values and beliefs are found attractive in a specific socio-cultural context, and thusly adopted, because they match some of the intuitive output of the radicalized mind. They probably do not generate the radicalization in the first place, and their lasting success is to be explained by the rich background of intuitions they meet. In essence, rather than ideology driving radicalization, radicalized individuals may be attracted to strict ideologies precisely because they are group-attracting and binding sets of ideas.

Scholars have questioned the importance and causal order of the adoption of extreme values or ideologies in the process of radicalization (e.g., Baez et al., 2017; Sageman, 2008). As we have alluded to above, empirical research suggests that knowledge of and commitment to extremist ideology often develops *after* an individual joins a group of radicalized militants (Ilardi, 2013). Radicalized neo-jihadi terrorists are noted as lacking a well-defined ideology and having an elementary understanding of Islam (Bartlett & Miller, 2012). A far-right militant insisted that ideology played an insignificant role in motivating his involvement. Instead, it equipped him with the means “to demonstrate [his] knowledge [in order to] impress others” (Horgan et al., 2017, p. 6). A former Neo-Nazi recalled that ideology was not a primary cause of his radicalization and “what mattered to [him] was that [he] would get a chance to participate in a real fight and kill the enemy” (Stern, 2014, p. 445). He followed up by saying that ideology is “just an excuse” for violence and “[others] become extremists—Nazis or join al-Qaeda—for the sake of justification.” Radicalized members of the IRA reported not knowing the political reasons for which they began fighting, calling into question the role of political ideology in radicalization (Bosi & Della Porta, 2012).

Ideological change is also not a pre-requisite nor associated with disengagement from terrorist groups (Altier et al., 2017). Despite their disengagement, individuals often continue to harbor radical political beliefs (Altier et al., 2017; Bjorgo & Horgan, 2008; Horgan, 2009). This could suggest that ideology might not be playing a premier role in the radicalization process. One reason ideological “packages” might be readily adopted by followers after being radicalized might reside in the fact that they provide “ready-made” sound justifications for violent actions. Such findings challenge the idea that disengagement needs to be preceded by a loss of faith in the ideology supposedly underpinning the radicalized behavior (Altier et al., 2017). Note also that radicalized individuals have been known to switch from one hostile ideology to another, even seemingly incompatible ones (e.g., from far left- to far-right extremism) (Koehler, 2019). A recent phenomenon of adherence to hybrid and incompatible ideologies has been referred to as “salad bar” extremism (Tiflati, 2022). In some of these instances, the decision to jump ship was influenced by the perception of the increasing costs and decreasing rewards of a specific membership. The late onset of ideological knowledge and its irrelevance to disengagement and group migration militate against ascribing an essential causal role to ideology in the process of radicalization. We are not the first ones to propose that ideology serves as post-hoc justification for one’s willingness to engage in violence rather than a cause of violence, per se (e.g., Baez et al., 2017; Baugut & Neumann, 2020; Bjorgo & Horgan, 2008; Borum, 2011a, b; Sageman, 2008, 2017a; Snow & Byrd, 2007; Speckhard & Ahkmedova, 2006; Webber & Kruglanski, 2017). We wish to argue yet a stronger claim that *most* reasons volunteered by agents for explaining their radicalization should be viewed skeptically. Too often the level of analytical reduction is too shallow to identify both the informational input and the structure of the processing mechanisms involved that give rise to the behaviors considered the hallmarks of radicalization.

## Discussion

**Fig. 1:**
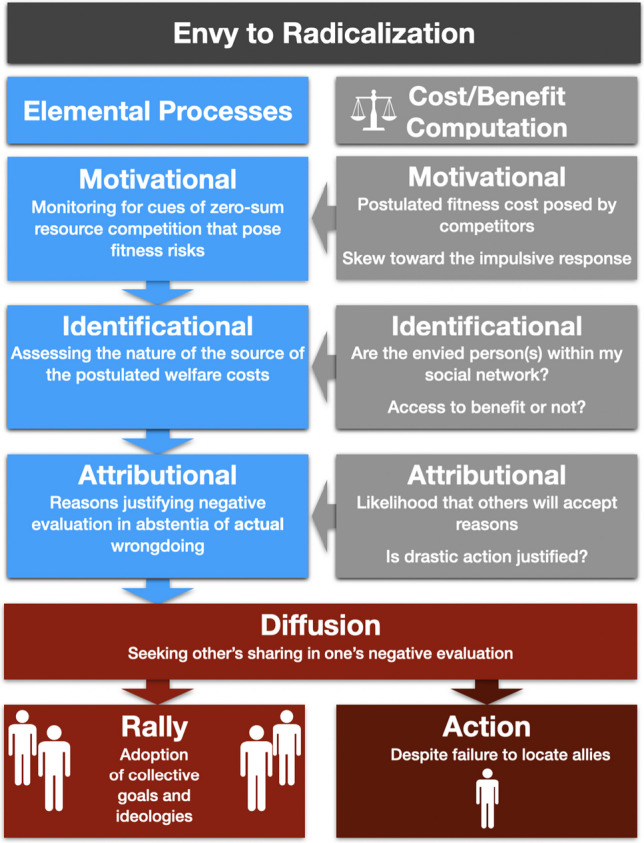
Graphical depiction of the process of envious militancy

Why did 4,000 citizens from EU Member States abandon the safety of their social environments to voluntarily join the fighting in foreign lands? Indeed, why have so many individuals in the West chosen the risky path of violence over non-violent political action? Envy parsimoniously accounts for characteristics of the process of radicalization and helps resolve the collective action problem we began the article with. The proposal weaves together the principal components we see playing a role in radicalization: envy and the deflection of responsibility, persuasion and diffusion of responsibility, and the alignment of interests for efficiency. Our approach is reductionist: we adopt the position that the functional structure of envy constitutes the core elements necessary, but not sufficient, to drive radicalization. Other motivations might be involved at distinct steps in the process depending on the conditions the individuals face, but those emotions do not explain the typical features that are parts of the basic architecture, sequence, and progression of the phenomenon. Figure 1 recaps the elemental processes of envious militancy discussed in the paper, along with some examples of the cost/benefit computations that might be involved.

A common objection to the envy hypothesis questions whether other emotions might serve as prime movers in radicalization. While further research is needed, theoretical analyses suggest that among the social emotions explored in the literature (e.g., anger, hatred, envy, disgust) (e.g., Landers & Sznycer, 2022; Sell et al., 2021; Sznycer & Lukaszewski, 2019; Sznycer et al., 2021; Tybur et al., 2013), only envy and hatred closely align with radicalization characteristics. For example, anger, associated with conspicuous signals for better future treatment (Sell & Lopez, 2020; Sell et al., 2021, 2022), does not match the stealthy tactics of radicalized individuals. Both envy and hatred share negative evaluations of a target’s impact on one’s fitness and a willingness to harm the target spitefully (Sell & Lopez, 2020; Sell et al., 2021), yet envy uniquely arises from perceived advantages of others and a zero-sum interpretation of the situation. Hatred, conversely, may be triggered downstream from envy’s evaluative components (Sell et al., 2021). We argue that envy, being proactive, motivates individuals to eliminate perceived social differentials and seeks to conceal itself by diffusing responsibility, aligning closely with key features of radicalization.

If our model has some validity, in agreement with Lankford (2018), the insistence on maintaining a distinction between loners and radicalized individuals belonging to collectives should be discarded. We see little difference in the process but for the fact that loners would be individuals who have not found a collective. While phenomena that appear similar to “radicalization” may occur in other contexts, such as genocides and mass killings (e.g., Mcdoom, 2020), we remain agnostic as to whether envy plays a central role or not in them. Furthermore, we do not hold that everyone in a terrorist group has been and is radicalized; there are numerous reasons for engaging in collective violence. However, within terrorist groups, some members are likely to be radicalized.

Why has radicalization been a problem in Western societies? Our liberal democracies facilitate social differentiation on the basis of merit. That differentiation is not countered with tribal-like “difference-crushing” leveling mechanisms. Indeed, free competitors succeed in enduringly differentiating themselves dramatically, generating some of the conditions that render envy salient. When such precipitating conditions are combined with stark divisions of the national body, where group differences in success and achievement exist, stereotyping and alignment with others in the same situation should be made easy. Therefore, militancy can quickly be ratcheted up. The coordination ease enhances the perception that a potential coalition that could provide support exists.

Following Davidson’s position (2001), while the acceptance of the reasons is more than enough for most day-to-day situations requiring social interaction, it should not be presumed that it shows us the way toward valid causal explanations. This is not to say that reasons and justifications should carry no weight, but that we need to be cautious when assessing such statements. For instance, as has been shown, the reasoning about envy-inspired actions previously perpetrated by deradicalized agents should betray their move to a new rationality and preference ranking (see Lienard & Moncrieff, 2023).

The hypotheses proposed in this model will need to be investigated ethnographically and experimentally in-depth. However, if accurate, the model challenges assumptions of existing programs aimed at combating radicalization that are currently part of State efforts for “preventing” or “countering” violent extremism. Many areas of activity of such programs, such as, for instance, costly programs targeting ideological change or educating youth about the dangers of violent extremism, are likely to be unsuccessful at reducing violent extremism. Efforts that would help to mitigate coalitional cleavages and promote an assimilationist agenda^10^ would probably be more successful. Also, addressing the “elephant in the room”—that self-interest and envy are core components of the radicalization process—would help. In line with our model, a deradicalization intervention provider in the UK notes how his method involves “[persuading] clients *to take responsibility* for their views or prejudices rather than blaming external factors” and “questioning why the Koran is being used as *justification* for domestic violence or the formation of a caliphate [emphasis added]” (Warrell, 2019).

The discussion of envy in this manuscript and its proposed role in radicalization opens avenues for further empirical examination. Unveiling the intricacies of envy’s role in radicalization necessitates innovative experimental approaches capable of dissecting envy at the functional level and the development of less obtrusive envy measures than currently available. Should the premise of the envy model hold, a number of hypotheses await empirical validation. For instance, the prevalence of envy is anticipated to be markedly elevated within radicalized cohorts compared to non-radicalized counterparts. Additionally, envy is posited to mediate individual differences (e.g., sex, impulsivity) and life history stages (e.g., early adulthood), correlated with a heightened risk of radicalization. As previously noted, envy is also likely to be imbricated with particular socioecological attributes (e.g., dominance-oriented hierarchies). The exploration of these hypotheses, among others, will advance our comprehension of envy’s role in radicalization and offer invaluable insights for developing interventions to prevent and counter radicalization and violent extremism.

## Data Availability

Not applicable.

## Footnotes

1 Other motivations might be involved at distinct steps in the process depending on the conditions that the individual faces, but those emotions are not necessary and do not explain the typical features that are parts of the basic architecture, sequence, and progression of the phenomenon.

2 The literature sometimes distinguishes between “benign envy” and “malicious envy”—in the former the agent competes whereas in the latter he aggresses. We conceptualize envy only as the malicious type which aligns with Cohen-Charash and Larson (2017) who argue that “envy, conceptualized and measured as a unitary construct, can lead to a wide range of reactions, both socially desirable and undesirable, depending on personal and situational moderators. We propose that the distinction between ‘benign’ and ‘malicious’ envy is unwarranted and advocate for the use of envy as a unitary construct.

3 In a zero-sum perspective, another individual’s success hurts (P. Boyer, personal communication, May 17, 2021). In fact, it literally does so. Envy activates the dorsal AAC where social pain is processed (Takahashi et al., 2009).

4 Sznycer advances the hypothesis of the existence of a psychology for social antagonism which predicts that in a situation where the agent conceives of his interactions with others as competitive, his social standing determines the core emotion that will drive his attitude (D. Sznycer, personal communication, May 21, 2021). As Fiske has proposed, one would envy up and scorn down (Fiske, 2011). Radicalization, we suggest, involves individuals in situations of lower standing, actual, assumed, or presumed.

5 I.e., the concern about the behavior’s legitimacy is in initiatory position for further downstream computation.

6 That is, the individual should become increasingly less the focus of positive social interactions, but more so, the destination of negative investments.

7 During the manuscript proofing stage, it came to our attention that a pertinent study by Knoll et al. (2022) on the nexus between envy and extreme violence has been recently published. This work provides an in-depth exploration of the mechanisms by which envy can alter perceptions, culminating in acts of extreme violence. While the insights from their study are not incorporated into the current manuscript, we would like to formally acknowledge the contributions of these authors to the academic discourse on this subject.

8 As one reviewer noted, the classification of incel violence as terrorism is disputed. Some argue that incels do not fit the criteria for violent extremism or terrorism (e.g., Cottee, 2021, p. 108; William, 2023, p. 53), while others see a substantial terrorism threat from violent incels (Hoffman et al., 2020).

9 Evolutionary models predict that males benefit more than females from offensive aggression (Lopez, 2017; McDonald et al., 2012; Tooby & Cosmides, 1988). That men are more likely to get radicalized to the point of committing violent acts is a consistent finding of the radicalization research (see *National Consortium for the Study of Terrorism and Responses to Terrorism (START)*, 2018; Pfundmair et al., 2019).

10 See for instance laws requiring the acquisition of local language skills in countries such as Switzerland.
